# Promising Therapy in Lung Cancer: Spotlight on Aurora Kinases

**DOI:** 10.3390/cancers12113371

**Published:** 2020-11-14

**Authors:** Domenico Galetta, Lourdes Cortes-Dericks

**Affiliations:** 1 Division of Thoracic Surgery, European Institute of Oncology, IRCCS, 20141 Milan, Italy; 2 Department of Biology, University of Hamburg, 20146 Hamburg, Germany; cortes-dericks@gmx.de

**Keywords:** Aurora kinases, AURKA, AURKB, lung cancer, lung cancer therapy

## Abstract

**Simple Summary:**

Lung cancer has remained one of the major causes of death worldwide. Thus, a more effective treatment approach is essential, such as the inhibition of specific cancer-promoting molecules. Aurora kinases regulate the process of mitosis—a process of cell division that is necessary for normal cell proliferation. Dysfunction of these kinases can contribute to cancer formation. In this review, we present studies indicating the implication of Aurora kinases in tumor formation, drug resistance, and disease prognosis. The effectivity of using Aurora kinase inhibitors in the pre-clinical and clinical investigations has proven their therapeutic potential in the setting of lung cancer. This work may provide further information to broaden the development of anticancer drugs and, thus, improve the conventional lung cancer management.

**Abstract:**

Despite tremendous efforts to improve the treatment of lung cancer, prognosis still remains poor; hence, the search for efficacious therapeutic option remains a prime concern in lung cancer research. Cell cycle regulation including mitosis has emerged as an important target for cancer management. Novel pharmacological agents blocking the activities of regulatory molecules that control the functional aspects of mitosis such as Aurora kinases are now being investigated. The Aurora kinases, Aurora-A (AURKA), and Aurora B (AURKB) are overexpressed in many tumor entities such as lung cancer that correlate with poor survival, whereby their inhibition, in most cases, enhances the efficacy of chemo-and radiotherapies, indicating their implication in cancer therapy. The current knowledge on Aurora kinase inhibitors has increasingly shown high potential in ensuing targeted therapies in lung malignancies. In this review, we will briefly describe the biology of Aurora kinases, highlight their oncogenic roles in the pre-clinical and clinical studies in lung cancer and, finally, address the challenges and potentials of Aurora kinases to improve the therapy of this malignancy.

## 1. Background

Lung cancer remains the most commonly occurring cancer attributed to the leading cause of cancer-related deaths globally. Non-small cell lung cancer (NSCLC) comprises 85% to 90% of lung cancers while small cell lung cancer (SCLC) represents 10–15% [1,2,3]. Survival varies according to the clinical stage ranging from 92% for earliest stage to 0% for advanced stage, respectively [4]. While surgery is the treatment of choice for early stages NSCLC, chemo-radiotherapy has been the therapy of choice for the advanced stages for many years [5]. Nowadays, in advanced non-small cell lung cancer, molecular targeted therapy is the standard first-line treatment for patients with identified driver mutations; on the other hand, chemotherapy (platinum-based) is the standard treatment for patients without driver mutation or those with unknown mutation status [6,7,8,9]. Moreover, immunotherapy has been now established as one of the most promising therapeutic options [10]. At this time, chemo- and radiotherapies are no longer the best option for this disease [11] so that further investigation of the molecular processes surrounding the oncogenesis of this malignancy warrants the development of more effective pharmacological agents.

The normal growth and development of a cell strictly depends on its ability to divide regularly during the various stages of cell division. This process is regulated by several mitotic kinases (serine-threonine kinases) which include polo-like kinases, cyclin dependent kinase 1 (CDK1), large tumor suppressor 1 (LATS1)-related kinases, never in mitosis (NIMA) -related kinases, and Aurora kinases execute critical roles in different stages of cell division [12].

The Aurora kinases consisting of three members termed Aurora-A (AURKA), -B (AURKB), and –C (AURKC) are complementary enzymes that modulate multiple mitotic events. These mitotic kinases are expressed in a cell cycle-dependent manner, most of which are activated in the G2/M phase being involved in mitotic chromosomal segregation [13,14]. These kinases bind to protein partners to be locally functional, that are tightly regulated in a temporal and spatial manner. AURKA and AURKB are the only ones of the Aurora kinase family to be expressed at detectable levels in somatic cells during mitotic processes and their function in cellular processes in the development of tumorigenic phenotypes has been extensively studied [15].

In NCSLC, overexpression of AURKA and AURKB has been associated with poor prognosis due to reduced overall survival [16,17,18,19,20,21,22,23,24] and both kinases have been found to either potentiate or repress chemo- and/or radiotherapy in lung cancer [1,25,26,27]. Some antimitotics that control microtubule dynamics such as vinca alkaloids, taxanes, and antineoplastic drugs targeting the regulatory mechanisms of mitosis [28] have shown effectivity in reducing the pro-oncogenic properties of Aurora kinases. Herein, we will present a comprehensive review of Aurora kinases in terms of their oncogenic actions in the development and treatment of lung cancer, and address the potential of targeting these molecules to improve the management of this neoplasm.

## 2. Cellular Localization and Functions of Aurora Kinases

**Figure 1:**
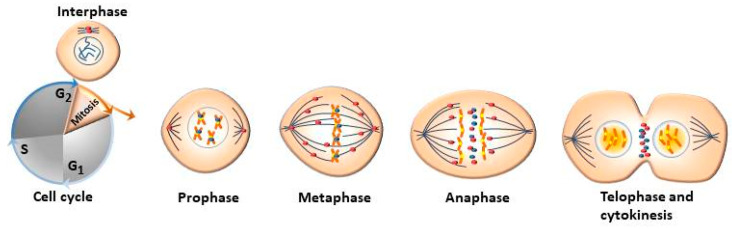
The Aurora kinases, AURKA (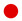) and AURKB (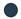) are expressed and activated from the G2 through the M phase being involved in mitotic chromosomal segregation [13,14]. AURKA is mainly localized in the centrosomes in interphase and mitotic cells, and is important for centrosome maturation and separation, which in turn are necessary for mitotic entry and bipolar spindle formation. AURKB is generally located at the kinetochore of mitotic cells and to the midbody of cytokinetic cells, which regulate kinetochore-microtubule attachments that are critical for chromosome alignment and segregation as well as to regulate cytokinesis [31,32]. AURKB and AURKC are thought to have the same distribution pattern in mitotic cells [33], hence, AURKC is not included in the diagram.

Aurora kinases have different subcellular distribution during the process of mitosis (Figure 1). AURKA and AURKB are constitutively present in mitotically active cells and are upregulated in highly proliferative tissues whereas the presence of AURKC is restricted to germ cells of both genders during meiosis [29]. The mitotic roles of the three Aurora kinases are thought to be dependent on their expression, temporal restriction, and localization, rather than on their kinase activity. Beside their localization, their functional differences are principally determined through binding with molecular partners and/or substrates [30].

The human AURKA is encoded by the AURKA gene located on chromosome 20q13.2 and is the most studied of Aurora kinase family. The kinase activity of AURKA and the levels of proteins linked to this kinase increase from G2 phase through the mitosis phase, reaching an activity peak during pro-metaphase. Its activation in the late G2 phase is required to trigger the initial activation of cyclin B1-CDK 1 at the centrosome and provide entry into mitosis [34]. Thr288 phosphorylation determines the activation of the kinase activity of AURKA while its deactivation is determined by the dephosphorylation of Thr288 by protein phosphatase 1 (PP1). The activity of AURKA is determined by a protein associated with microtubules targeting protein for xenopus kinesin-like protein (TPX2), which triggers its autophosphorylation and protects it from the inhibitory property of PP1 [12,35]. The organization of the anaphase spindle at the end of mitosis is determined by the presence of AURKA, whose abundance is downregulated by the proteolysis process mediated by the APC/C-Cdh1-dependent proteasome [13].

Aurora-B is a protein encoded by the AURKB gene found on chromosome 17p13.1 [36]. AURKB guarantees a correct kinetochore–microtubule attachment thanks to the collaboration of other substrates such as survivin, borealin, and the internal centromere protein (INCEP). In fact, AURKB phosphorylates INCEP directly, increasing its kinase activity in vitro [37] In mitotic cells, AURKB is localized to the kinetochore of prometaphase chromosome, contributing to their proper alignment at metaphase, and to the spindle midzone of anaphase cells and execute important actions for the completion of cytokinesis reviewed in [38]. Aurora-B can cause defects in chromosome separation and cytokinesis, implying its pivotal role in cell division [31]. At the end of mitosis, both AURKA and AURKB are subjected to ubiquitination and proteasome degradation after dephosphorylation by PP2A or PP1 [39].

The Aurora-C is encoded by the AURKC gene localized at chromosome 19q13.43 (Gene ID: 6795). AURKB and AURKC are thought to have similar distribution pattern sequences, substrates such as INCENP [40], surviving, and borealin, and functions during cell division [33]. The only detectable difference is the localization of AURKC in interphase germ cells that involves the centrosomes rather than the nuclei [41].

## 3. Pre-Clinical Studies on Aurora Kinases

### 3.1. Overexpression of AURKA and AURKB Correlates with Unfavorable Therapeutic Response in Lung Cancer

Aurora kinases are often overexpressed in lung cancer correlating with profound reduction in favorable therapeutic response in lung cancer therapy. In several studies, AURKA has been proven to be overexpressed in NSCLC and associated with decreased patient survival. For instance, Liu and colleagues [16] found increased expression of AURKA in NSCLC tumor samples that correlated with decreased time to progression and overall survival. Using bio-informatic-based enrichment analyses, Zhang’s group [17] reported that elevated mRNA of AURKA, CDC20, and TPX2 were significantly associated with poor prognosis in smoking-related lung adenocarcinoma, indicating the potential of using AURKA, CDC20, and TPX2 as biomarkers for predicting poor prognosis in smoking-related lung cancer.

Recently, the expression levels of mitotic spindle genes (Aurora kinases (AURKA, AURKB, AURKC), kinesin-like protein 11 (KIF11), discs large-associated protein 5(DLGAPS5), cytoskeleton-associated protein 5 (CKAP5), monopolar spindle 1 kinase (TTK) and β-tubulins (TUBB and TUBB3), microtubule nucleation factor (TPX2), and their association with the clinicopathological characteristics were investigated in NSCLC tumor and adjacent normal lung tissues. Al-Khafaji and colleagues demonstrated that, except AURKC, all other genes examined showed increased RNA gene expression in NSCLC tumor tissues compared to normal lung tissues, and only AURKA was significantly associated with a poor prognosis in NSCLC patients. This finding has shown that AURKA inhibitors can give an advantage in terms of survival in patients with high levels of AURKA [18].

In the study of Schneider and colleagues [19], five specific mitosis-associated genes: AURKA, DLGAPS5, TPX2, KIF11, and CKAP5 were investigated to elucidate their correlation to prognosis in NSCLC patients. These authors affirmed that all of the tested genes were highly amplified in NSCLC samples compared to the corresponding normal lung tissues and were associated with poor survival. Notably, AURKA was found as a statistically significant negative prognostic marker. In a separate work, the prognostic value of AURKA, Ki67, p53, p21, and WAF1 in resected NSCLC tissues showed that positive expressions of AURKA, Ki67, and p53 were unfavorable factors in the prognosis of NSCLC patients. Accordingly, the overexpression of AURKA was found as an independent adverse factor associated with shorter overall survival in NSCLC and, thus, may serve as a prognostic indicator in this tumor [20]. Not only is the overregulation of AURKA in NSCLC patients was correlated with poorer overall survival and progression-free interval but was also implicated in cisplatin-resistance in lung cancer cells. This AURKA-dependent chemoresistance was reversed upon silencing of AURKA confirming its contribution to a reduced drug response to conventional chemotherapy [21].

In contrast to the above reviewed studies, Lo lacono et al. [14] found that upregulated levels of AURKA in NSCLC biopsies as compared to corresponding normal lung tissues showed no correlation with patient survival and that the observed overexpression is restricted to specific subtypes and poorly differentiated lung cancers.

Similar to AURKA, upregulated levels of AURKB in lung cancer have also been associated with negative prognosis and poor therapeutic response. As reported by Yu and colleagues [42], overmodulation of AURKB significantly correlated with reduced overall survival and disease-free interval in NSCLC patients. They also noted that there was an over amplification of AURKB in NSCLC with impaired response to CCDP (cisplatin) and paclitaxel. In corroboration of this observation, silencing of AURKB resensitized NSCLC cells to these chemotherapeutic agents by establishing correct chromosome segregation and restoring p53 expression, which suggests AURKB as a promising therapeutic target in this malignancy.

Cumulative data from immunohistochemical analyses of NSCLC samples indicated that high AURKB expression correlated with poor prognosis [22,23,24], lymph node metastasis, poor tumor differentiation grade, histological type of NSCLC [24,43] and genetic instability [44], suggesting that AURKB may contribute to malignancy and may represent a valid target in NSCLC. Conversely, Al-Khafaji’s team [45] measured extensive overexpression of AURKB mRNA in frozen NSCLC tissues— the highest being in squamous carcinoma, but this showed weak associations with higher pathological stages and, in contrast to previous reports, showed no correlation with overall survival.

Malignant pleural mesothelioma (MPM) is a rare, aggressive cancer of the pleural surface with poor prognosis [46,47]. In human mesothelioma tissues, Crispi’s group [48] found upregulated levels of AURKA and related genes as well as increased amounts of both AURKA and AURKB in five mesothelioma cell lines indicating a potential involvement of Aurora kinases in the oncogenesis of MPM. In another study, microarray analyses of pleural mesotheliomas revealed that the more aggressive mesotheliomas expressed increased levels of AURKA and AURKB and functionally related genes involved in cell cycle control and mitosis [49].

A summary of the effects of overexpressed AURKA and AURKB in lung cancer is depicted in Table 1.

### 3.2. Impact of AURKA and AURKB in Chemo- and Radiotherapy in Lung Cancer

A growing amount of evidence has shown that Aurora kinases could either augment or repress drug response in lung cancer treatment. It has been recently shown that residual disease and acquired resistance to epidermal growth factor receptor (EGFR) inhibitors required AURKA activity. The suppression of this kinase activity via Aurora inhibitors prevented the adaptive survival program, thereby increasing the degree and duration of EGFR response in preclinical models [25]. A separate study has demonstrated that AURKB activation was associated with acquired resistance to EGFR tyrosine kinase inhibitors (EGFR TKIs) in NSCLC. These EGFR-resistant cells were sensitive to AURKB inhibitor barasertib and S49076, which reduced the levels of pH3—a major product of AURKB mediating G1/S arrest and polyploidy [1].

AURKA/nuclear factor kappa-light-chain-enhancer of activated B cells (NF-κB) signaling has been associated with radio-resistance in human lung adenocarcinoma. Liu and colleagues [50] found that up-modulated AURKA was responsible for in vitro radio-resistance of docetaxel-resistant SPC-A1/DTX lung adenocarcinoma cells (SPC-A1/DTX). In this work, NF-κB was identified as a downstream target of AURKA in SPC-A1/DTX cells linking NF-κB signaling with radio-resistance in human lung adenocarcinoma docetaxel-resistant cells. Within this context, the teams of Linardopoulos [51] and Sun [52] demonstrated that A549 lung cancer and SKOV ovarian cancer cells were resistant to cytotoxic agents such as adriamycin and VP-16 (etoposide). However, upon treatment with an AURKA inhibitor, NF-κB activity was downregulated and the efficacy of cytotoxic drugs was improved. These findings affirmed that inhibition of AURKA could enhance the potency of chemotherapeutic agents and is capable of reversing the acquired resistance resulting from activated NF-κB.

Studies indicate that p53 status is one of the contributing factors of chemosensitivity in tumor cells. According to Wu’s report [26], A549 lung cancer cells with wild type p53 exhibited low levels of AURKA and were sensitive to treatments with gefitinib. However, intriguingly, silencing of p53 in these cells showed high expression of AURKA rendering the cancer cells insensitive to gefitinib-induced apoptosis thus, indicating a role for AURKA in gefitinib resistance. Accordingly, this study showed that silencing of AURKA in p53-knockdown cancer cells sensitized the A549 cancer cells to gefitinib, which also proved the inhibitory action of p53 in decreasing the ability of AURKA in conferring resistance to gefitinib.

Orth and colleagues, using the cancer genome atlas (TCGA) showed that, in a specific cohort of patients with lung adenocarcinoma, high levels of AURKA and TPX2 were associated with improved overall survival in response to taxane-based radiochemotherapy, but not in case of non-taxane-based radiochemotherapy, chemo- or radiotherapy only manifesting a AURKA/TPX2 dependent paclitaxel-mediated radiosensitization that led to improved OS in lung adenocarcinoma patients [27].

The modulatory activity of AURKB on taxane response in NSCLC has been recently reported. Al-Khafaji’s team [45] found a paradoxic correlation between the expression levels of AURKB, patient survival, and taxane sensitivity in NSCLC. In this study, the overexpression of AURKB reduced survival in chemotherapy of naïve patients but had a beneficial effect in patients under taxane regimens. In particular, low levels of AURKB was found associated with taxane resistance. Silencing of AURKB validated this concept, which resulted in a marked increased resistance to paclitaxel, confirming the effect that reduced AURKB expression modulates insensitivity to taxane in human lung cancer cells and, thus, may serve as predictive parameter to taxane response in NSCLC.

An overview of the impact of AURKA and AURKB on chemo- and/or radiotherapy in lung cancer is shown in Table 2.

### 3.3. Inhibition of AURKA and AURKB Suppresses Their Pro-Tumorigenic Actions in Lung Cancer

Both AURKA and AURKB have been implicated in the development of lung cancer growth. Because of this, several specific Aurora kinase inhibitors (AKIs) have been developed and have provided promising results in preclinical settings.

A current study has functionally characterized the AURKA inhibitor, TC-A2317, in human lung cancer cells. This inhibitor could slow the cell proliferation of lung cancer cells by causing an aberrant formation of centrosome, microtubule spindles, and prolonging mitosis. Depending on the cell type, TC-A2317 treatment caused apoptosis, autophagy, or senescence at the cellular level [53].

The preclinical characterization of the AURKA inhibitor, R763/AS703569 showed that it could inhibit Aurora kinases along with other kinases including FMS-related tyrosine kinase 3 (FLT3) conferring a potent anti-proliferative ability against many cell types having enlarged cell size, endo-reduplication, and apoptosis. In vivo, R763/AS703569 was able to inhibit tumor growth in different tumors including lung cancer [54]. In another study, the combined treatment with valproic acid (VPA), belonging to histone deacetylase inhibitors and the Ras inhibitor, farnesylthiosalicylic acid (FTS), salirasib synergistically reduced the proliferation of A549 lung cancer cells, DLD1 colon, and ARO thyroid carcinoma cells by downregulating Ras and blocking the expression of survivin and AURKA [55].

The traditional herbal medicine, tanshinone including tanshinone 1 (T1), tanshinone llA (T2A), and cryptotanshinone (CT) is believed to inhibit the growth of lung cancer cells in vitro. Indeed, Ma and colleagues [56] presented evidence that tanshinones could restrain NSCLCs by suppressing AURKA via upregulation of miR-32 expressions and other related miRNAs.

According to Yu’s study [57], the novel protein, suppressed in lung cancer (SLAN), also known as KlAA0256 is under-expressed in lung cancer tissues. Mechanistically, this protein could negatively modulate AURKA activity by directly repressing its kinase activity or reducing the levels of AURK and, importantly, restrain cell proliferation of human lung cancer cells indicating the therapeutic potential of SLAN in AURKA-associated cancer development.

The use of molecular-targeted agents during radiotherapy of NSCLC is most likely an effective strategy to avoid repopulation, thereby increasing therapeutic effects. Sak and colleagues [58] showed that inhibition of AURKB with low AZD1152-hydroxyquinazoline pyrazol anilide (AZD 1152-HQPA) concentrations concomitant to radiotherapy negatively affects repopulation of NSCLC cell lines, indicating the combined efficacy of AURKB inhibition and irradiation in regrowth of lung cancer cells. In another study, varying concentrations of AZD 1152-HQPA conferred a reduction in cell viability and ensued apoptotic cell death in four malignant mesothelioma cell lines, but without considerable effect on the level of AURKB expression at the translational level [59].

AZD 1152, another selective inhibitor of AURKB, was also able to inhibit the growth of different human cancer xenografts including that of the lung, via suppression of histone H3 phosphorylation, accumulation of 4N DNA in cells and increased proportion of polyploid cells. These cellular effects may serve as useful indicators for assessing the therapeutic efficacy of AZD 1152 [60].

Survivin, a member of the inhibitor of apoptosis gene family, binds to AURKB to form the chromosome passenger complex, both of which are overexpressed in many tumors. Kim et al. [61] found that dual inhibition of survivin by an antisense oligonucleotides and AURKB inhibitor ZM447439 synergistically radiosensitized mesothelioma cells by promoting mitotic cell arrest in mesothelioma cells after irradiation.

S49076, a MET/AXL/FGFR inhibitor has been shown to negate AURKB activity and improve the anti-tumor efficacy of radiotherapy. S49076 could exert its cytotoxic action at low doses on Met-dependent cells, GTL16 and U87-MG, via MET inhibition, whereas it restrained the growth of MET-independent lung adenocarcinoma cells H441, H460, and A549 at higher but clinically relevant doses by targeting AURKB. Additionally, S49076 was able to improve the efficacy of radiotherapy in both MET-dependent and -independent cell lines under in vitro and in vivo conditions [62].

Another anti-tumor inhibitor, quercetin, showed effective inhibition of AURKB pro-tumorigenic activities by directly binding to AURKB under in vitro and in vivo conditions in JBC Cl41 and A549 lung cancer cells and A549 xenografts, respectively [63]. Likewise, HOl-07 [(*E*)-3-((*E*)-4-(benzo[*d*][1,3]dioxol-5-yl)-2-oxobut-3-en-1-ylidene) indolin-2-one] has been found to specifically inhibit AURKB in lung cancer cells in vitro and A549 xenografts via inhibition of histone H3-Ser 10 phosphorylation in a dose- and time-dependent manner. Notably, silencing of AURKB profoundly reduced the sensitivity of tumor cells to HOl-07 that further attested the inhibitory activity of HOl-07 to AURKB [64].

An interesting study found that ceftriaxone, an FDA-approved cephalosporin antibiotic, primarily used against pneumonia, meningitis, and gonorrhea exhibited an off-target effect as it also conveyed an anti-tumor activity in vitro and in vivo. Ceftriaxone decreased anchorage-independent cell growth by targeting AURKB in lung adenocarcinoma cancer cell lines and effectively diminished the growth of A549 and H1650 lung tumor xenografts by inhibiting AURKB [65].

The major component of rice bran oil, 24-methylenecyloartanyl ferulate (24-mCAF) was found capable of inhibiting cell proliferation and activating apoptosis in A549 lung cancer cells through an upregulation of the Myc binding protein 1A (MYBBP1A), a tumor suppressor that represses cancer development. 24-mCAF was also able to restrain the activity of AKT, AURKB, and two Ser/Thr kinases involved in MYBBP1A modulation, demonstrating the potential of these proteins as therapeutic targets in NSCLC [66].

Recently, Dos Santos et al. [67] investigated the relationship among KRAS, AURKA, and AURKB in lung cancer cells and evaluated the role of inhibition of Aurora kinases can lead to real therapeutic benefit. These authors demonstrated that using the dual AURKA and AURKB inhibitor, AI II, reduces growth, viability, and anchorage-independent growth in a KRAS-dependent manner, showing that AURKA and AURKB are promising targets for KRAS-induced lung cancer therapy.

An investigational topoisomerase inhibitor, daurinol, in combination with radiation could effectively decrease lung cancer growth in xenograft mouse models through the inhibition of AURKA and AURKB [68]. Likewise, ZM447439, an Aurora kinase inhibitor, has shown effective inhibition of both AURKA and AURKB activities via reduction in histone H3 phosphorylation, resulting in decreased cell growth in mesothelioma cell lines [48].

GSK 1070916, a potent inhibitor for AURKB and AURKC, has demonstrated anti-tumor growth capacity in a broad range of tumors. This inhibitor could repress cell proliferation by conferring failure in cell division leading to polyploidy and apoptosis. In vitro assays showed that GSK 1070916 conferred a dose-dependent inhibition of histone H3 phosphorylation, a specific substrate of AURKB and conveyed anti-tumor effects in ten human tumor xenografts, including lung cancer [69].

VX-680 is a pan-Aurora kinase inhibitor that still has insufficient clinical efficacy. Using a genome-wide CRISPR screen, Huang and colleagues [70] identified genes whose reduction ensues synthetic lethality with VX-680. According to this study, synthetic lethal interaction between Haspin (a serine/threonine kinase that phosphorylates histone H3 during mitosis) depletion and VX-680 was mediated by the inhibition of Haspin and AURKB but not AURKA. This work underscores that the combined inhibition of Haspin and AURKB could have a better efficacy than using a single agent treatment, as has been observed in both head and neck squamous cell carcinoma and NSCLC.

An overview of the therapeutic effects of AURKA and/or AURKB inhibition in lung cancer is depicted in Table 3.

## 4. Aurora Kinase Inhibitors in Clinical Studies

Alisertib (MLN8238) is an investigational selective inhibitor of AURKA. As single-agent, the safety and activity of alisertib has been evaluated in a Phase II study in advanced refractory or relapsed tumors, including non-small cell lung cancer (NSCLC), small lung cancer (SCLC), head and neck squamous cell carcinoma (HNSCC), breast cancer, and gastro-esophageal adenocarcinoma, all exhibiting high levels of AURKA. In this investigation, a manageable safety profile was observed among all of the tumors tested characterized by fatigue, alopecia, anemia, neutropenia, and diarrhea. Alisertib conferred objective partial responses in patients with advanced breast cancer (9 (8%) of 49 patients) and SCLC (10 (21%) of 48 patients), indicating an antitumor activity in these tumors that warrants further assessment; particularly, combining alisertib with other therapeutic agents [71].

Godwin and colleagues [72] assessed whether the combination of an epidermal growth factor receptor (EGFR) inhibitor, erlotinib, and alisertib exerts a synergistic action in wild-type EGFR NSCLC in a phase I/II clinical trial. Erlotinib is an oral reversible tyrosine kinase inhibitor (TKI) that targets EGFR, known to have efficacy in NSCLC. In patients with recurrent or metastatic EGFR wild-type NSCLC, the combination of alisertib and erlotinib was tolerable and an antitumor activity was noted. The maximum tolerated dose (MTD) was found at DL3 (E 150 mg daily + A 40 mg BID). The recommended sequential phase II dosing study is now being assessed.

Owonikoko and colleagues [73] evaluated in a phase II trial of patients with relapsed or refractory SCLC the effectiveness and safety of paclitaxel plus alisertib versus paclitaxel plus placebo as second-line treatment. The objectives of this study were the evaluation of (a) progression-free survival, and (b) the impact of c-Myc protein expression and genetic alterations on clinical outcomes. In this work, a modest efficacy signal as second-line therapy was observed with alisertib/paclitaxel in relapsed or refractory SCLC. The predictive potential of c-Myc expression and cell cycle mutations as biomarkers for alisertib efficacy is encouraging, but necessitates further validation.

In a prospective, phase II, open-label, multi-institutional study, danusertib was adopted as single agent for treating patients with different advanced cancers including SCLC (n = 18) and NSCLC (n = 56) as second line treatment. The end-points of this trial were (a) to evaluate the anti-tumor activity of danusertib; (b) to evaluate the safety profile of this drug monitoring its pharmacokinetics (PK). Results of this trial showed that danusertib as single agent reached only marginal anti-tumoral activity, while its safety and PK profile was consistent with previous experience [74]. Another phase II study evaluated the efficacy of danusertib in advanced/metastatic NSCLC using progression-free survival at four months (PFS-4) in a Simon two-stage design. The expression of AURKA-B, TPX-2, MDR, Scr, and survivin by immunohistochemistry (IHC) and amplification of FGFR1 by fluorescence in situ hybridization (FISH) on tumor biopsies was also evaluated. Although the histological analyses by IHC and (FISH) were not yet included in this report, the authors concluded that danusertib showed limited activity, which is insufficient to meet the predefined threshold to call efficacy in NSCLC when administered as monotherapy at the dose/schedule of 500 mg/m^2^ as 24 h IV infusion q2w. Nevertheless, this study showed a manageable safety profile of danusertib [75].

AT9283, an inhibitor of AURKA and AURKB, has been assessed in a phase I dose escalation study in patients with advanced solid tumors including NSCLC. AT9283 was generally tolerated with toxic effects of reversible dose-related myelosuppression, gastrointestinal disturbance, fatigue, and alopecia. AT9283 was well tolerated up to the MTD of 27 mg/m^2^/72h. Pharmacodynamic analyses showed antiproliferative and anti-apoptotic actions of AT9283. No objective tumor responses were observed; however, four patients with esophageal (n = 1), colorectal (n = 1), and NSCLC (n = 2) demonstrated prolonged stable tumor disease of ≥6 months based on Response Evaluation Criteria in Solid Tumors (RECIST) [76].

A summary of the Aurora kinase inhibitors tested in clinical studies is shown in Table 4.

## 5. Conclusions and Perspectives

Encouraging pre-clinical and clinical data have led to the development of novel anti-mitotic agents to augment standard management of lung cancer. Phase I/II clinical studies with Aurora kinase inhibitors alisertib and danusertib, alone and combination with other standard chemotherapeutic agents, have demonstrated increased antitumor effects that necessitate further clinical assessment. The overexpression of AURKA and AURKB, which correlates with poor therapeutic outcomes, implies a role in oncogenesis in lung cancer, strengthening the idea of targeting specific molecules in such cases. Importantly, each type of Aurora kinase should be treated as an autonomous target, alone and in combination, due to different expression levels and treatment responses [77]. As well as being a factor in conveying resistance to conventional chemotherapy, both AURKA and AURKB might be useful as biomarkers in predicting therapeutic response. Although Aurora kinases are promising therapeutic targets in several types of cancer, including lung cancer, a major issue is distinguishing normal cells from malignant cells. Normal cells require the physiological function of Aurora kinases; therefore, inhibition may result in high toxicity. Targeting Aurora kinases is likely to be a double-edged sword [29]. Thus, a better understanding of the fundamental properties and associated molecular partners/substrates of Aurora kinases is needed to reveal their precise function. Doing so should provide new insight into the discovery of target molecules and therapeutic strategies in treatment of lung cancer.

DG and LCD equally conceived, drafted and wrote the manuscript. LCD approved the final version of the manuscript.

## Figures and Tables

**Table 1:** Effects of overexpression of AURKA and/or AURKB in lung cancer.

Aurora Kinase	Prognostic/Oncogenic Effect	Experimental Specimen; Method	Reference
AURKA	Correlates with decreased OS	NSCLC tumor samples; IHC	[16]
AURKA	Potential biomarker for predicting poor prognosis in smoking-related LC	Cancer Genome Atlas data base; bioinformatic-based analysis	[17]
AURKA	Independent predictor of poor prognosis	NSCLC tumor and adjacent normal lung tissues; PCR	[18]
AURKA	Correlates with poor survival	NSCLC tumor and corresponding normal lung tissues; IHC	[19]
AURKA	Correlates with shorter OS	NSCLC tissues; IHC	[20]
AURKA	Correlates with poor OS and progression-free interval; contributes to reduced drug response	NSCLC tissues; IHC	[21]
AURKA	No correlation with patient survival	NSCLC tumor and corresponding normal lung tissues; IHC and RT-PCR	[14]
AURKA	Potential pro-oncogenic role in MPM	MPM tissues; qPCR analysis	[48]
AURKB	Correlates with reduced OS and disease-free interval; impairs response to cisplatin and paclitaxel	NSCLC tumor biopsies; RT-PCR	[42]
AURKB	No correlation with OS	Frozen NSCLC tissues; qPCR	[45]
AURKB	Correlates with poor prognosis	NSCLC tissues; IHC Same as above NSCLC tumor and adjacent lung tissues; IHC	[22] [23] [24]
AURKB	Correlates with lymph node metastasis and poor tumor differentiation grade	NSCLC tissues; IHC NSCLC tumor and adjacent lung tissues; IHC	[43] [24]
AURKA and AURKB	Potential pro-oncogenic role in MPM	MPM tissues and cell lines; transcript analysis	[48]
AURKA and AURKB	High expression levels in aggressive MPM	MPM tissues; microarray analysis	[49]

Abbreviations: NSCLC, non-small cell lung cancer; LC, lung cancer; IHC, immunohistochemistry; RT-PCR, reverse transcription polymerase chain reaction; qPCR, quantitative polymerase chain reaction; MPM, malignant pleural mesothelioma; OS, overall survival.

**Table 2:** Impact of AURKA and AURKB on chemo- and/or radiotherapy in lung cancer.

Aurora Kinase	Effect on Chemo-and/or Radiotherapy	Experimental Sample; Method	Reference
AURKA	Resistance to EGFR inhibitors; triggers disease recurrence	EGFR-mutant lung adenocarcinoma cells; in vitro/in vivo analysis	[25]
AURKA	Overexpression confers resistance to radiotherapy in docetaxel-resistant, SPC-A1/DTX lung adenocarcinoma cells	SPC-A1 lung cancer cells; in vitro analysis	[50]
AURKA	AURKA/TPX2 overexpression associates with paclitaxel-mediated radiosensitization; improves OS	Cancer genome atlas lung adenocarcinoma cohort; in vitro analysis	[27]
AURKA	Resistance to gefitinib-induced apoptosis	A549 lung cancer cells: in vitro analysis	[26]
AURKA	Resistance to cytotoxic agents such as adriamycin and VP-16 (etoposide)	A549 lung cancer cells; in vitro analysis	[51,52]
AURKB	Resistance to EGFR inhibitors	NSCLC tumor samples; in vitro analysis	[1]
AURKB	Low levels confer resistance to paclitaxel	NSCLC cell lines; in vitro analysis	[45]

Abbreviations: NSCLC, non-small cell lung cancer; EGFR, epidermal growth factor receptor; SPC-A1, human lung cancer cell line; DTX, docetaxel; TPX2, targeting protein for Xklp2; OS, overall survival.

**Table 3:** Effects of specific inhibition of AURKA and/or AURKB in lung cancer.

Inhibitor	Therapeutic Effect	Reference
24-mCAF (major component of rice bran oil)	Inhibition of cell proliferation and activation of apoptosis in A549 lung cancer cells	[66]
S49076 (MET/AXL/FGFR inhibitor)	Inhibition of AURKB confers improved radiotherapy in MET-dependent and MET–independent lung cancer cell lines	[62]
TC-A2317 (AURKA inhibitor)	Reduction in cell proliferation in lung cancer cells; induction of cell type-dependent apoptosis and autophagy	[53]
Quercetin	Direct binding to AURKB inhibits its pro-tumorigenic actions in lung cancer cells and lung cancer xenografts	[63]
AKII (dual AURKA and AURKB inhibitor)	Repression of cell growth, viability and induction of apoptosis in a KRAS-dependent manner in A549 and H358 lung cancer cells	[67]
Tanschinone (traditional herbal medicine)	Inhibition of lung cancer cells in vitro by suppression of AURKA	[56]
HOI-07	Attenuation of cancer cell-anchorage-independent growth and inhibition of lung cancer xenografts through suppression of AURKB and histone H3 Ser10 phosphorylation	[64]
Ceftriaxone (cephalosporin antibiotic)	Off-target effect on AURKB; decreases anchorage-dependent growth of lung cancer cells; growth reduction in lung cancer xenografts	[65]
AZD 1152-HQPA (inhibitor of AURKB) concomitant to radiotherapy	Prevents repopulation of NSCLC cells during radiotherapy	[58]
Valproic acid and FTS (RAS inhibitor)	Reduction in A549 cell proliferation; block expression of survivin and AURKA	[55]
SLAN (KIAA0256)	Repression of AURKA kinase activity and expression levels; inhibition of cell proliferation of lung cancer cells	[57]
R763/AS703569	Exerts anti-proliferative activity via inhibition of Aurora kinases and FMS-related tyrosine kinases; growth inhibition of lung tumor xenografts	[54]
AZD 1152	Selective inhibition of AURKB; growth inhibition of lung tumor xenografts	[60]
ZM447439 (AURKB inhibitor) and survivin antisense oligonucleotide	Sensitization of mesothelioma cells to radiotherapy	[61]
AZD 1152-HQPA	Reduction in cell viability; induction of cell apoptosis in mesothelioma cell lines	[59]
ZM447439	Inhibition of AURKA and AURKB; suppression of cell growth of mesothelioma cells via reduction in histone H3 phosphorylation	[48]
Daurinol (topoisomerase inhibitor)	In combination with radiation decreases growth of lung cancer xenografts through inhibition of AURKA and AURKB	[68]
GSK 1070916 (AURKB and AURKC inhibitor)	Dose-dependent inhibition of histone H3 phosphorylation causing failure in cell division and polyploidy leading to apoptosis: tumor growth suppression in murine model	[69]

Abbreviations: NSCLC, non-small cell lung cancer; FTS, farnesylthiosallicylic acid, salirasib); SLAN, suppressed in lung cancer; HOl-07, [(E)-3-((E)-4-(benzo[*d*][1,3]dioxol-5-yl)-2-oxobut-3-en-1-ylidene) indolin-2-one], AZD 1152-HQPA, AZD1152-hydroxyquinazoline pyrazol anilide; 24-mCAF, 24-methylenecyloartanyl ferulate; MET, encodes receptor tyrosine kinase c-MET for hepatocyte growth factor; AKII, Aurora kinase inhibitor II.

**Table 4:** Aurora Kinase Inhibitors in Clinical Studies.

Inhibitor	Therapeutic Effect/Clinical Trial	Reference
Alisertib (MLN8238), AURKA inhibitor	Antitumor effect in advanced SCLC/phase II study	[71]
Combined alisertib and erlotinib, EGFR inhibitor	Synergistic antitumor effect in wild- type EGFR NSCLC/phase I/II study	[72]
Combined alisertib and paclitaxel	Modest efficacy signal as second-line therapy in relapsed or refractory SCLC/phase II study	[73]
Danusertib (PHA-739358), pan-Aurora kinase inhibitor	Marginal antitumor action after failure of prior systematic therapies in NSCLC and squamous NSCLC/phase II study	[74]
Danusertib	Insufficient efficacy as monotherapy in advanced/metastatic NCSLC/phase II study	[75]
AT9283, AURKA and AURKB inhibitor	No objective tumor response; stable RECIST disease of ≥6 months/phase I study	[76]

Abbreviations: NSCLC, non-small cell lung cancer; SCLC, small cell lung cancer; EGFR, epidermal growth factor receptor; RECIST, response evaluation criteria in solid tumors.
